# Metabolic Changes Associated with a Rat Model of Diabetic Depression Detected by Ex Vivo ^1^H Nuclear Magnetic Resonance Spectroscopy in the Prefrontal Cortex, Hippocampus, and Hypothalamus

**DOI:** 10.1155/2018/6473728

**Published:** 2018-04-08

**Authors:** Kun Liu, Liangcai Zhao, Wen Xu, Qiuting Lin, Yongjin Zhou, Xiaoyan Huang, Xinjian Ye, Jiawei He, Guanghui Bai, Zhihan Yan, Hongchang Gao

**Affiliations:** ^1^Radiology Department of the Second Affiliated Hospital and Yuying Children's Hospital, Wenzhou Medical University, Wenzhou 325035, China; ^2^School of Pharmaceutical Sciences, Wenzhou Medical University, Wenzhou 325035, China

## Abstract

Diabetic patients often present with comorbid depression. However, the pathogenetic mechanisms underlying diabetic depression (DD) remain unclear. To explore the mechanisms underpinning the pathogenesis of the disease, we used ex vivo ^1^H nuclear magnetic resonance spectroscopy and immunohistochemistry to investigate the main metabolic and pathological changes in various rat brain areas in an animal model of DD. Compared with the control group, rats in the DD group showed significant decreases in neurotransmitter concentrations of glutamate (Glu) and glutamine (Gln) in the prefrontal cortex (PFC), hippocampus, and hypothalamus and aspartate and glycine in the PFC and hypothalamus. Gamma-aminobutyric acid (GABA) was decreased only in the hypothalamus. Levels of the energy product, lactate, were higher in the PFC, hippocampus, and hypothalamus of rats with DD than those in control rats, while creatine was lower in the PFC and hippocampus, and alanine was lower in the hypothalamus. The levels of other brain metabolites were altered, including N-acetyl aspartate, taurine, and choline. Immunohistochemistry analysis revealed that expressions of both glutamine synthetase and glutaminase were decreased in the PFC, hippocampus, and hypothalamus of rats with DD. The metabolic changes in levels of Glu, Gln, and GABA indicate an imbalance of the Glu-Gln metabolic cycle between astrocytes and neurons. Our results suggest that the development of DD in rats may be linked to brain metabolic changes, including inhibition of the Glu-Gln cycle, increases in anaerobic glycolysis, and disturbances in the lactate-alanine shuttle, and associated with dysfunction of neurons and astrocytes.

## 1. Introduction

As a chronic and progressive disease, diabetes is commonly associated with several neuropsychiatric comorbidities, such as depression, dementia, schizophrenia, and bipolar disorder [1]. The incidence of depression is two to three times higher in patients with diabetes than in healthy persons [2, 3]. Depression is also one of the most neglected symptoms in patients with diabetes and is directly linked with reduced quality of life, lower medical adherence, poor nutrition, lower physical and mental wellbeing, and even higher mortality [4–6]. Studies examining the pathogenesis of diabetic depression (DD) may contribute to developing novel interventions or therapeutic targets.

The mechanisms unpinning DD remain unclear. However, oxidative stress has been suggested as an important underlying mechanism on the basis of results from pathophysiology studies [7–9]. Oxidative stress causes significant changes in the function of several macromolecules (proteins, lipids, and DNA), and the persistent effects of these changes are related to the etiology of diabetes and depression. Hypothalamic-pituitary-adrenal axis dysregulation also plays crucial roles in the pathogenesis of DD [8]. In addition, DD may be attributed to impaired neurogenesis and brain-derived neurotrophic factor synthesis [8]. Despite these findings, more questions than answers regarding the mechanisms underpinning DD remain.

Studies have shown that depression may be induced by unbalanced glutamatergic and GABAergic neurotransmitter metabolism. An in vivo magnetic resonance spectroscopy (MRS) study has reported that, compared with those in healthy participants, the glutamate (Glu) and the glutamate and glutamine (Glx) levels were significantly lower in the anterior cingulate in patients with major depression [10]. Gamma-aminobutyric acid (GABA) and Glx levels were also decreased in the prefrontal regions of patients with depression, and Glu and glutamine (Gln) levels were decreased in the subcortical area in type 2 diabetic patients with major depression [11, 12]. These suggest a possible role of decreased glutamatergic and GABAergic neurotransmission within these brain regions in the pathogenesis of depression. However, studies have also shown that increased glutamatergic and GABAergic neurotransmission may be involved in depression. Xu et al. found that the Glx/creatine value was significantly increased in the thalamus in patients with bipolar depression compared with that in healthy controls [13]. Glodzik-Sobanska et al. revealed an elevation of GABA levels in the frontal lobes of poststroke patients with depression [14]. These inconsistencies in the changes of glutamatergic and GABAergic neurotransmission may stem from several factors, including the poor sensitivity of in vivo MRS, other pathophysiological conditions of the patients, and differences in the brain regions investigated.

Usually, ex vivo ^1^H nuclear magnetic resonance (NMR) spectroscopy is conducted at higher field strength than in vivo ^1^H NMR spectroscopy. Hence, the detection sensitivity, regional specificity, and dispersion of metabolite peaks are remarkably improved in ex vivo ^1^H NMR spectroscopy [15]. One of our previous studies done by this approach found that hyperbilirubinemia can result in region-specific perturbation of metabolic pathways including neurotransmitter transition and energy metabolism in the brain [15]. These results have contributed to the understanding of the pathogenesis of bilirubin encephalopathy. Thus, we used this same approach in the present study along with pathological analyses of key proteins to investigate the features of metabolism in samples of the prefrontal cortex (PFC), hippocampus, and hypothalamus obtained from rats modeling DD. The purpose of the present study was to explore metabolic variations in these brain regions, which may provide important clues for understanding the mechanisms of DD or for identifying novel interventions.

## 2. Materials and Methods

### 2.1. Animals

Male Wistar rats (about 6 weeks of age, weighing 163.4 ± 3.9 g) were purchased from the SLAC Laboratory Animal Co. Ltd. (Shanghai, China). The animals were allowed to adapt to the laboratory environment for 1 week before the experiments. During the whole experimental process, rats were kept in a temperature- and humidity-controlled environment on a 12 h light/dark cycle. Food and water were available ad libitum. All animals were treated in strict accordance with the National Institutes of Health's *Guide for the Care and Use of Laboratory Animals*.

### 2.2. Rat Model of DD

The induction of DD was conducted according to a previously described procedure [16, 17]. The animals were randomly allocated to the control group (*n* = 8) or the DD group (*n* = 18). After a 12 h fast, rats in the DD group were injected intraperitoneally with streptozotocin (Sigma, St. Louis, MO, USA) dissolved in 0.01 mol/L citric acid solution (pH = 4.5) at a single dose of 64 mg/kg. Control rats received injections of citrate buffer alone. Seventy-two hours after streptozotocin administration, blood glucose was measured after obtaining blood through tail nicking with a Glucotrend monitor (Roche Diagnostics, Switzerland). The rats with increased blood glucose level (≥16.70 mmol/L) were subjected to the chronic unpredictable mild stress (CUMS) procedure. The stress procedure consisted of a range of stressors, including crowding (10 rats cohabiting in one cage for 24 h), cage tilt (40° for 24 h), white noise (80 ± 2 db for 6 h), wet bedding (24 h), cold swim (4°C for 5 min), stroboscopic illumination (300 flashes/min for 6 h), and tail pinch (for 1 min). Over a period of 28 days, one of these stimuli was randomly chosen and used to the rats in case the rats were capable of anticipating the stimulus. For each rat, every stimulus was applied four times within 28 days. Control animals were housed in a separate room and had no contact with the stressed group. Body weight was measured on days 0, 14 (2 weeks), and 28 (4 weeks) after the CUMS procedure.

### 2.3. Open-Field Test

The open-field test is an evaluation of spontaneous and exploratory activities as well as of anxiety-like behavior in a novel environment in rodents [16, 18]. The rats in the DD group were subjected to this test after the 4-week CUMS procedure. The open-field apparatus was made of opaque materials. Its bottom was 90 × 90 cm square and divided into 25 equilateral squares. The height of the wall surrounding the bottom was 40 cm. Being placed in the central square, the rat was permitted to explore freely for 5 min. A video-tracking system was used to record the distances traveled and the time spent in the central and peripheral areas. After each rat finished the test, the apparatus was cleaned with 100% alcohol and dried so that the next rat would not be influenced by the smell of the preceding rat. A behavioral analysis system was used to analyze the acquired video-tracking data.

### 2.4. Morris Water Maze Test

Spatial learning and memory were tested using the Morris water maze [19]. The Morris water maze apparatus was a water tank (110 cm in diameter, 30 cm high) with a platform (7 cm in diameter) inside the tank (raised 19 cm from the tank bottom) that was not visible to the rats. The tank was filled with 20 cm height of water maintained at a temperature of 24–26°C. The rats were trained for 4 consecutive days, and on each training day, the rats swam four 60 s trials (initial placement rotated for each trial) or until they reached to the hidden platform. On the fifth day, the platform was removed for a 60 s trial and the swim path as well as the time spent swimming were recorded by a video-tracking computer system.

### 2.5. Sample Collection

After the behavioral tests were completed, 14 rats in the DD group and 6 control rats were decapitated and the brain was rapidly removed. The bilateral PFC, hippocampus, and hypothalamus were quickly dissected, frozen in liquid nitrogen, and stored at −80°C until they were treated for use in ^1^H NMR studies. The remaining rats were anesthetized with 4% chloral hydrate and perfused intracardially with phosphate-buffered saline (PBS) followed by 4% paraformaldehyde in PBS. Then, brain tissues were collected, fixed in 10% formalin for 24 h, imbedded in paraffin, and stored at 4°C until used for immunohistochemical studies.

### 2.6. Brain Metabolite Extraction and Acquisition of ^1^H NMR Spectra

Our previously reported method was used to prepare brain tissue extracts and acquire ^1^H-NMR spectra [15, 20]. Briefly, the tissue was weighed into a centrifuge tube. Then, ice-cold methanol (4 mL/g) and distilled water (0.85 mL/g) were added into the tube, homogenized by vortex at 4°C. Subsequently, chloroform (2 mL/g) and distilled water (2 mL/g) were added to the tube and mixed by vortex. After being kept on ice for 15 min, the final mixture was centrifuged at 1000 ×g for 15 min at 4°C. The supernatant was extracted and lyophilized for approximately 24 h. The dried metabolite mixture was weighted and then dissolved in 0.6 mL of 99.5% D_2_O for NMR analyses.

A Bruker Avance III 600 MHz NMR spectrometer was used to conduct all ^1^H NMR experiments. The crucial parameters were set as follows: spectral width, 12,000 Hz; acquisition time, 2.65 s per scan; relaxation delay, 10 s; and number of scans, 256. By using the TopSpin software (v2.1 pl4, Bruker BioSpin, Germany), all spectra were preprocessed with a reference of the lactate peak (CH_3_, 1.33 ppm) and manual correction of the phase and baseline.

### 2.7. Data Processing of NMR Spectra and Multivariate Pattern Recognition

For exploiting whole metabolic information in the spectra, AMIX software package was used to divide all NMR spectra into integral regions, which had an equal width of 0.01 and 0.0015 ppm. Due to the residual peak from the suppressed water resonance, the integral of the NMR region (*δ* 5.85–4.60) was set to zero. Other integral regions were normalized to the total sum of the spectral intensity. Then, the normalized integral values were entered into SIMCA-P+ 12.0 software (Umetrics, Umeå, Sweden) as variables and were mean-centered for multivariate data analysis. The projection to latent structures-discriminant analysis (PLS-DA) was used to discriminate the class and identify the biomarkers [21]. In order to acquire the most efficient 2-D representation of the information, the principal component (PC) score plot of the first two principal components (PC1 and PC2) was applied to visualize the data. Each point on the PC score plot represented an individual spectrum of the sample [15]. The position of each point along a given axis in the score plot was influenced by variables in the same axis in the loading plot [22]. PLS-DA revealed differences in the composition of different groups, which were necessary to eliminate outliers and enhance the quality of the PCA model. Differences in the metabolites between groups were shown as coefficient of variation plots, which facilitate interpretation because the loadings resemble NMR spectra. The scores and loading plots complemented each other. The loading plot—wherein differential peaks of metabolites are shown as positive and negative signals suggest the relative metabolite changes—was applied to determine which spectral variables were the main contributors to the discrimination of the samples on the score plot [15, 20, 23].

### 2.8. Immunohistochemistry

For immunohistochemical staining, tissue sections were cut to a thickness of 3 *μ*m, deparaffinized with xylene, and rehydrated in a graded series of ethanol. Immunohistochemistry was performed according to procedures described in previous studies [24, 25]. Briefly, fixed brains were incubated with glutamine synthetase (GS) (1 : 200, Santa Cruz Biotechnology, CA) and glutaminase (GLS) (1 : 200, Abcam, ab156876) antibodies overnight at 4°C. The sections were washed in PBS 3 times, then incubated with horseradish peroxidase-conjugated secondary antibodies at 37°C for 1 h, and finally terminated by 3,3′-diaminobenzidine. The images were photographed with a Nikon Eclipse 80i (Nikon, Japan). For each immunohistochemical stain, the signal densities were analyzed using three selected sections from the PFC, hippocampus, and hypothalamus with Image-Pro Plus software (version 6.0, Rockville, MD, USA).

### 2.9. Target Metabolic Changes in Tissues and Statistical Analysis

By reference to internal trimethylsilyl-propionic-2,2,3,3-d4 acid, the concentrations of metabolites were determined from the spectra and expressed as mmol/kg wet tissue weight. Significant statistical difference between the rats modeling DD and the control rats for the identified metabolites, body weight, and behavioral data was determined using Student's *t*-test with SPSS software (version 13.0, SPSS Inc., USA). The level of statistical significance was set at *p* < 0.05.

## 3. Results

### 3.1. Body Weights and Behavioral Analyses

**Figure 1:**
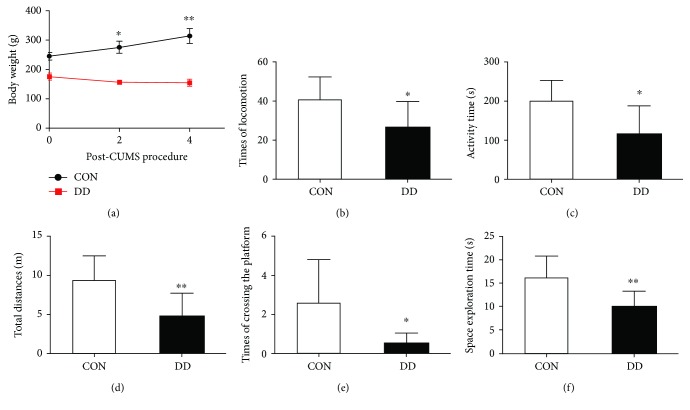
Changes in body weight, performance on the open-field test and Morris water maze test for rats in the control (CON) and DD groups. (a) body weight, (b) times of locomotion, (c) activity time, (d) total time, (e) times of crossing the platform, (f) space exploration time. Significant level: ^∗^*p* < 0.05, ^∗∗^*p* < 0.01.

Compared with the control group, rats in the DD group showed a significant decrease in body weight on days 0, 14, and 28 after the CUMS procedure (Figure 1(a)).

In the open-field test, the total distance traveled by rats in the DD group was significantly lower than that traveled by rats in the control group. A reduction in activity time and time spent in locomotion indicated less spontaneous activity in these rats (Figures 1(b)–1(d)). These data suggested that rats treated with streptozotocin and CUMS had less interest than control rats in exploring a new environment.

In the Morris water maze test, the number of times the rats crossed the location where the platform had been hidden and the amount of time spent in the platform quadrant were significantly different between the two groups (Figures 1(e) and 1(f)). Compared with rats in the control group, rats in the DD group traveled irregularly during the 1 min probe test, with significantly fewer crossings of the area where the platform had been located and less time spent in the platform quadrant. This suggested that after 4 days of training, rats in the DD group appeared to perform worse in learning and consolidating the platform location, which can be interpreted as an expression of memory impairment in these rats.

### 3.2. ^1^H NMR Spectra and PLS-DA Analysis

**Figure 2:**
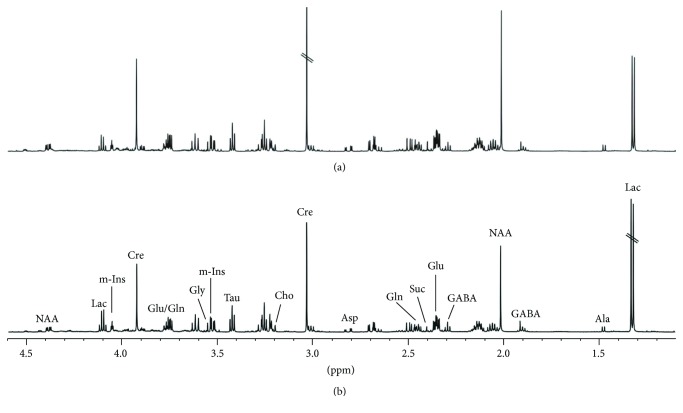
Representative ^1^H NMR spectra of the PFC extracts obtained from rats in the control (a) and DD (b) groups.

Figure 2 shows representative ^1^H NMR spectra of the PFC extracts obtained from rats in the DD and control groups. Assignments presented in Figure 2 are based on our previous publication and were verified by 2D ^1^H-^1^H COSY and TOCSY spectra (data not shown) [20]. Many endogenous metabolites can be measured from the ^1^H NMR spectra, including lactate (Lac), Glu, Gln, GABA, aspartate (Asp), succinate (Suc), creatine (Cre), alanine (Ala), N-acetyl aspartate (NAA), taurine (Tau), myo-inositol (m-Ins), glycine (Gly), and choline (Cho).

**Figure 3:**
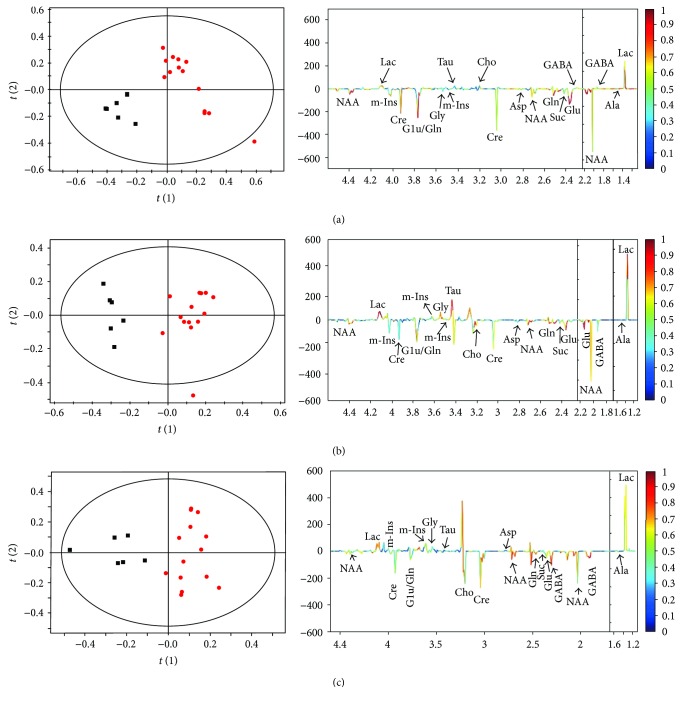
PLS-DA scores (left) and coefficient-coded loading plots (right) for the models discriminating the rats in the DD group (red dots) from control rats (blank squares) for data obtained in PFC (a), hippocampus (b), and hypothalamus (c) samples.

After the segmentation of the NMR spectra of PFC extracts, a multivariate data analysis was used to determine metabolic profile changes in rats in the DD group. Marked separation was found between the DD and control groups in the *t*(1) direction on PLS-DA score plots (Figure 3), which indicates a different spectral feature of the two groups in the PFC. The corresponding coefficient-coded loading plot (Figure 3) showed that Glu, Lac, NAA, and Cre contribute to the separation. Similar patterns of metabolism were found in the score plots and loading plots of the spectra from the hippocampal and hypothalamic extracts (Figure 3).

### 3.3. Quantitative Analysis of Metabolic Alterations

**Figure 4:**
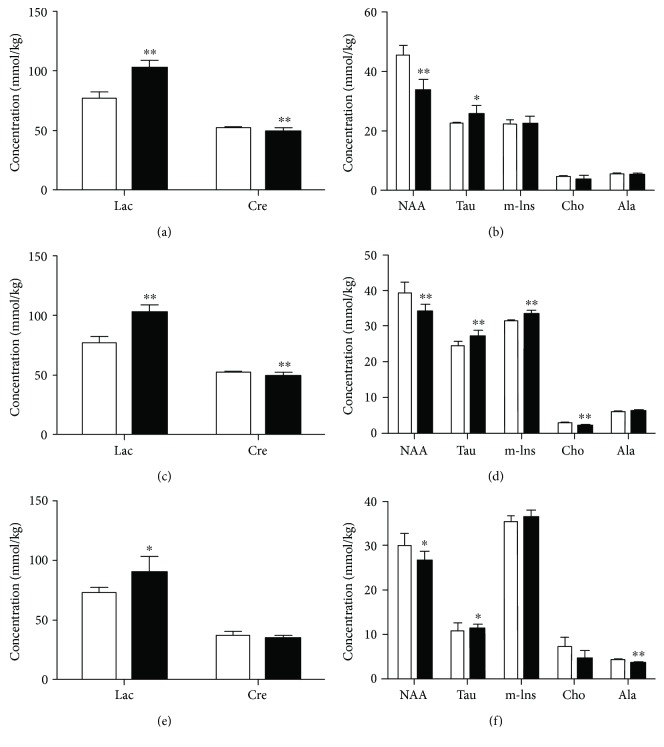
Changes in concentrations of energy and other brain metabolites in the PFC (a, b), hippocampus (c, d), and hypothalamus (e, f) in the control (white bars) and DD (black bars) groups. Significant level: ^∗^*p* < 0.05, ^∗∗^*p* < 0.01.

**Figure 5:**
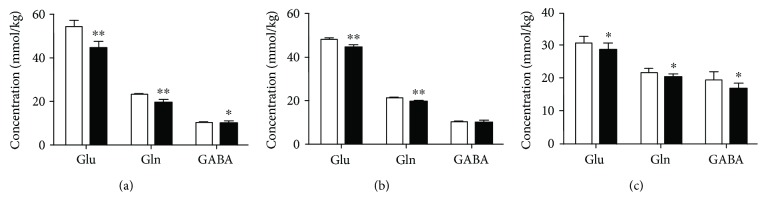
Changes in concentrations of glutamate (Glu), glutamine (Gln), and GABA in the PFC (a), hippocampus (b), and hypothalamus (c) in the control (white bars) and DD (black bars) groups. Significant level: ^∗^*p* < 0.05, ^∗∗^*p* < 0.01.

The changes in the metabolites of the PFC, hippocampus, and hypothalamus obtained from rats in the DD group are shown in Figures 4 and 5. DD induced a significant increase in Lac but no apparent change in Suc in the PFC, hippocampus, and hypothalamus. Cre was decreased in the PFC and hippocampus of these rats. NAA was significantly decreased in the PFC, hippocampus, and hypothalamus, and Cho was decreased in the latter two brain areas, while Tau was increased in the former two regions. Ala was decreased only in the hypothalamus, and m-Ins was increased only in the hippocampus. Marked decreases in Glu and Gln neurotransmitter concentrations were found in the hippocampus of rats in the DD group. Similar decreases in these neurotransmitters along with Asp and Gly were found in the PFC and hypothalamus. GABA was decreased only in the hypothalamus.

### 3.4. Key Enzymes in the Glu-Gln Cycle

**Figure 6:**
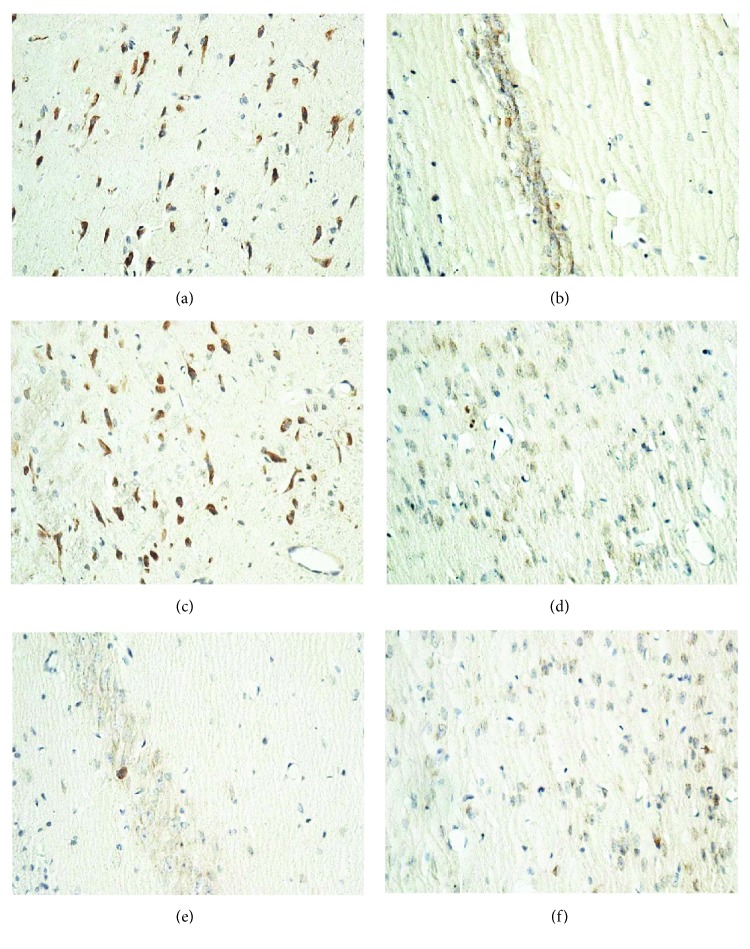
Immunohistochemistry of glutamine synthetase (GS) in the PFC, hippocampus, and hypothalamus of rats in the control (a, b, c) and DD (d, e, f) groups.

**Figure 7:**
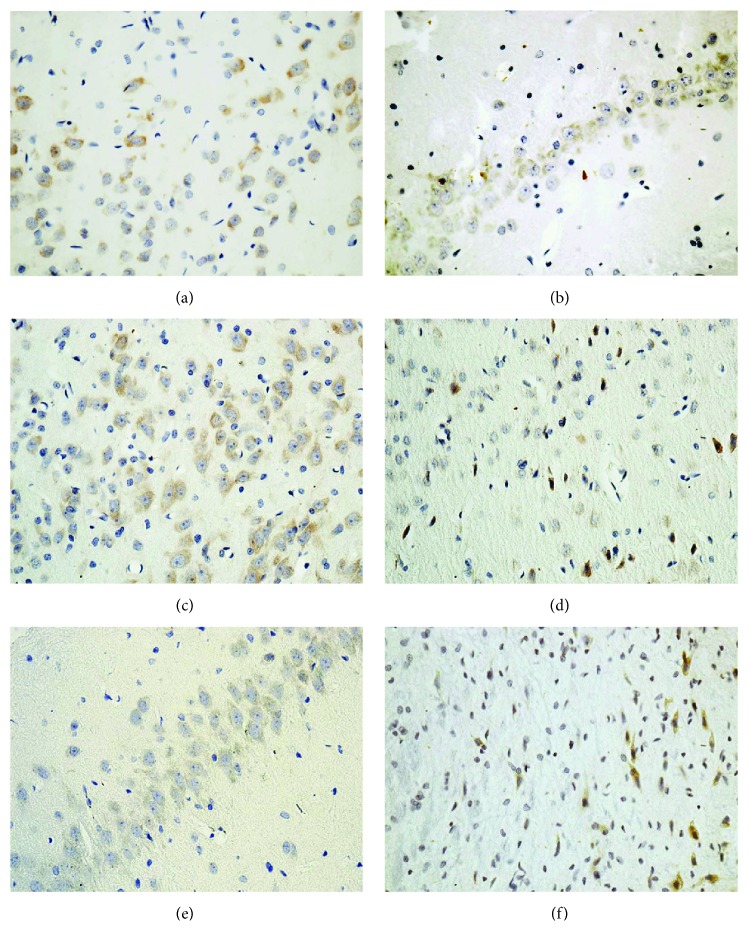
Immunohistochemistry of glutaminase (GLS) in the PFC, hippocampus, and hypothalamus of rats in the control (a, b, c) and DD (d, e, f) groups.

As a crucial enzyme in the cytoplasm of astrocyte, GS results in the formation of Gln from Glu [24]. GLS is an important enzyme in neuronal axons and generates Glu from Gln. Immunohistochemical staining showed that GS was decreased in the PFC, hippocampus, and hypothalamus of rats in the DD group, indicating inhibition of the pathway from Glu to Gln (Figure 6). Decreased labeling of GLS neurons was found in these brain areas (Figure 7), which was in accordance with the reduction of Gln to Glu.

## 4. Discussion

In this study, we successfully established a rat model of DD and comprehensively reported metabolic alterations in the PFC, hippocampus, and hypothalamus in these rats as determined by ex vivo ^1^H NMR spectroscopy. We found marked metabolic changes of neurochemicals containing some crucial neurotransmitters and energy products, as well as disruption in the markers of neuronal and astrocytic activity in these brain areas in rats modeling DD.

### 4.1. Establishment of the Rat Model of DD

Streptozotocin is frequently used to induce diabetes in rats [26]. After an adequate dose of streptozotocin is injected, rats generally maintain a high level of blood glucose and fail to gain body weight. Near the end of the present study, rats in the DD group exhibited approximately half the body weight of control rats. The CUMS procedure is thought to simulate unpredictable, stressful daily life events [27]. It has a significant impact on anxiety and metabolism and results in anhedonia, which is a major symptom of human depression. The CUMS procedure is commonly used to develop depression in rats. In our study, rats in the DD group subjected to the CUMS procedure exhibited significant decreases in locomotion and in exploratory activity in the open-field test, representing both a loss of interest in new stimulating situations and a deficit in motivation. In the Morris water maze test, rats in the DD group spent less time in the target quadrant and had fewer crossings over the hidden platform, indicating impaired memory function and spatial learning deficits after the CUMS treatment. Taking together, the body weight change and the results of the behavioral tests indicated the successful establishment of a rat model of DD.

### 4.2. Changes in Neurotransmitter Metabolism

As an important metabolic cycle between astrocytes and neurons, the Glu-Gln cycle regulates homeostasis of neurotransmitters such as Glu, Gln, and GABA in the brain. Glu is the main excitatory neurotransmitter, and GABA is the main inhibitory neurotransmitter. In this study, both Glu and Gln levels were significantly decreased in the PFC, hippocampus, and hypothalamus of rats modeling DD, which is consistent with previous studies showing a significant reduction of Glu and Gln levels in the subcortical region of the brain in patients with type 2 diabetes and major depression [12]. Glu and Gln were significantly increased in the frontal region of diabetic patients [28], but were significantly decreased in the PFC and hippocampus of depressed rats [29]. We speculated that the changes of Glu and Gln in our study is likely due to the effect of depression. Compared with that in controls, GABA was significantly decreased only in the hypothalamus of the rats modeling DD. This is similar to the previous observation that major depression is associated with reduced GABA levels in the dorsomedial/dorsal anterolateral PFC of the brain [11]. Decreased GABA was also observed in the hippocampus of diabetic rats [30]. Both depression and diabetes result in the decreased GABA in human and rat brain. Thus, it cannot be concluded whether the observed change of GABA in our study is rather due to diabetes or depression. Since the metabolic pathways which regulate the producing and the cycling of Glu, Gln, and GABA are closely coupled, alterations in these neurotransmitters reflect change of the balance of the Glu-Gln shuttling between astrocytes and neurons in the PFC, hippocampus, and hypothalamus of rats modeling DD. This perhaps resulted from decreased Glu synthesis in neurons via GS and/or reduced Glu uptake by astrocytes, where Glu is transformed into Gln by GLS. Interestingly, our immunohistochemical staining showed that both GS and GLS were attenuated in the PFC, hippocampus, and hypothalamus of rats in the DD group. The decreases in Glu and Gln were in agreement with the alterations of these key proteins in the Glu-Gln cycle.

The reduced levels of Glu and Gln also suggested reduced precursor pools, which may have been caused by diabetes-induced impairment of glucose metabolism and depression-induced dysfunction of astrocyte. Studies have revealed that changed astrocyte metabolism is necessary for maintaining cerebral Glu and GABA levels in diabetic rats and impaired astrocyte function is responsible for the reduction of Glu and Gln concentrations in depressed patients [31, 32].

Depression may be ascribed to a change in the function of glutamatergic and GABAergic neurons. Glu and GABA play crucial roles in the regulation of emotion and stress [33]. Glu is released from the presynaptic cell and acts on postsynaptic receptors, such as N-methyl-D-aspartate (NMDA) receptors [34]. GABA acts at inhibitory synapses in the brain by binding to specific transmembrane receptors, including GABA_A_ and GABA_B_ receptors [35, 36]. It has been demonstrated that the expression levels of NMDA and GABA receptors are changed in synaptic densities in the brains of diabetic rats [37, 38]. However, the relationship between DD and the changes in Glu and GABA levels as well as NMDA and GABA receptor activity remains ambiguous and needs further study.

### 4.3. Changes in Energy Metabolism

Brain energy supply relies almost entirely on glucose oxidative metabolism in the mitochondria. When brain energy requirements exceed the oxidative metabolism rate, the anaerobic glycolysis pathway will be enhanced and the production of Lac will be markedly increased. Lac will then be used as the energy substrate to maintain normal cerebral energy homeostasis and metabolism [39]. In this study, the increased Lac level may indicate that anaerobic glycolysis was increased and the mitochondrial function was impaired in the PFC, hippocampus, and hypothalamus of rats modeling DD. Similarly, an increased Lac level is associated with diabetes in humans [23]. Thus, the change of Lac in our study is likely due to the effect of diabetes. Besides, the lactate-alanine shuttle is responsible for nitrogen exchange in the brain of mammals. Compared with control rats, Lac was increased and Ala was decreased in the present study, suggesting a disorder in the lactate-alanine shuttle of the hypothalamus in the DD group. Cre also takes part in the regulation of cellular energy metabolism and is utilized as an energy reservoir in cells with a high energy demand [40]. Compared with that in control rats, Cre was significantly decreased in the PFC and hippocampus but not in the hypothalamus of rats in the DD group. However, Cre was significantly increased in the ventral lateral prefrontal region of bipolar depressed patients [41] and also in the hippocampus of diabetic rats [42]. This suggested that the observed reduction of Cre in our study only occurs when diabetes and depression appear together and may be the result of a combined effect of diabetes and depression. In addition, the change of Cre could be considered as one of the specific changes in DD. The changes in Cre also reflect abnormal energy metabolism in the PFC and hippocampus of rats in the DD group.

### 4.4. Changes in Other Metabolites

NAA is regarded as a surrogate marker of the status of the neuronal function. Sharp decreases in the levels of NAA were found in the PFC, hippocampus, and hypothalamus of rats in the DD group. Similarly, a significant reduction in NAA has been reported in patients with depression [43] and also in patients with diabetes [28, 44]. Thus, it cannot be concluded whether the reduction of NAA in our study is due to diabetes or depression. Our results indicated that neuronal dysfunction occurred in rats of the DD group.

As markers of astrocytic activity, Tau, m-Ins, and Cho have crucial functions in regulating the intracellular osmolarity in astrocytes [45, 46]. Disturbances in their levels have been interpreted as a compensatory response to an increase in intracellular osmolarity in glial cells [47]. In our study, brain region-specific changes of these metabolites were observed in rats in the DD group. For example, Tau was increased in the PFC and hippocampus and m-Ins was increased in the hippocampus, while Cho was decreased in the hippocampus and hypothalamus. Both depression and diabetes result in the increased Cho in human brain [41, 48]. Hence, the decreased Cho in our study only occurs when diabetes and depression appear together and may be the result of a combined effect of diabetes and depression. In addition, the decreased Cho may be one of the specific changes in DD. Both depression and diabetes cause the increased Tau in rat hippocampus [29, 49], while depression causes the decreased Tau in rat PFC [50]. Thus, it cannot be concluded whether the observed change of Tau in our study is due to diabetes or depression. The level of m-Ins in the hippocampus was found increased in the animal model of diabetes [23, 49], but decreased in the animal model of depression [29], suggesting that diabetes plays a dominant role in the change of m-Ins in this study. The changes of Tau, m-Ins, and Cho are likely mechanisms compensating for changed astrocytic osmolarity in the PFC, hippocampus, and hypothalamus.

## 5. Conclusion

The current study was designed to detect changes of numerous metabolites in the rat brain to explore the mechanisms underpinning DD. Changes in the metabolism of the neurotransmitters Glu, Gln, GABA, Asp, and Gly and the energy products Lac and Cre as well as other metabolites in the PFC, hippocampus, and hypothalamus were observed. Our results suggested that DD may be associated with metabolic changes, such as inhibition of the Glu-Gln cycle, increase in anaerobic glycolysis, and disruption of the lactate-alanine shuttle, as well as with the dysfunction of neurons and astrocytes. Our results will advance the understanding of the underlying mechanisms of DD.
